# Sodium nitroglycerin induces middle cerebral artery vasodilatation in young, healthy adults

**DOI:** 10.1113/EP087022

**Published:** 2018-06-13

**Authors:** Jenna M. Schulz, Baraa K. Al‐Khazraji, J. Kevin Shoemaker

**Affiliations:** ^1^ School of Physical Therapy, Department of Health Sciences Western University London ON Canada; ^2^ Department of Physiology and Pharmacology Western University London ON Canada; ^3^ School of Kinesiology, Department of Health Sciences Western University London ON Canada

**Keywords:** cerebral blood flow, magnetic resonance imaging, middle cerebral artery, nitroglycerin, transcranial Doppler

## Abstract

**New Findings:**

- **What is the central question of this study?**
  Nitric oxide causes dilatation in peripheral vessels; however, whether nitric oxide affects basal cerebral artery dilatation has not been explored.
- **What is the main finding and its importance?**
  This study demonstrated that vasodilatation occurs in the right middle cerebral artery in response to exogenous nitric oxide. However, blood velocity decreased and, therefore, overall cerebral blood flow remained unchanged. This study provides new insight into the role of nitric oxide in cerebral blood flow control.

**Abstract:**

Recent evidence indicates that basal cerebral conduit vessels dilate with hypercapnia, with a nitric oxide (NO) mechanism explaining one way in which parenchymal cerebral arterioles dilate. However, whether NO affects basal cerebral artery dilatation remains unknown. This study quantified the effect of an exogenous NO donor [sodium nitroglycerin (NTG); 0.4 mg sublingual spray] on the right middle cerebral artery (rMCA) cross‐sectional area (CSA), blood velocity and overall blood flow. Measures of vessel CSA (7 T magnetic resonance imaging) and MCA blood velocity (transcranial Doppler ultrasound) were made at baseline (BL) and after exogenous NTG or placebo (PLO) administration in young, healthy individuals (*n* = 10, two males, age range 20–23 years). The CSA increased in the rMCA [BL, 5.2 ± 1.2 mm^2^; PLO, 5.4 ± 1.5 mm^2^; NTG, 6.6 ± 1.5 mm^2^, *P *< 0.05; mean ± SD]. Concurrently, rMCA blood velocity decreased from BL during NTG compared with PLO (BL, 67 ± 10 cm s^−1^; PLO, 62 ± 10 cm s^−1^; NTG, 59 ± 9.3 cm s^−1^, *P *< 0.05; mean ± SD]. However, total MCA blood flow did not change with NTG or PLO [BL, 221 ± 37.4 ml min^−1^; PLO, 218 ± 35.0 ml min^−1^; NTG, 213 ± 46.4 ml min^−1^). Therefore, exogenous NO mediates a dilatory response in the rMCA, but not in its downstream vascular bed.

## 1 INTRODUCTION

Optimal cerebral blood flow (CBF) is achieved through changes in perfusion pressure (Nagata et al., 2016) and adjustments in cerebrovascular conductance (Aaslid, 1992). The control of cerebral microvasculature has received considerable attention (Hamel, 2005), whereas the role of the large conduit cerebral arteries in CBF control and its distribution in conscious humans has only recently gained research focus. Early data obtained in larger animal studies (Mchedlishvili, Mitagvaria, & Ormotsadze, 1973; Yoshida, Meyer, Sakamoto, & Handa, 1966) and literature reviews (Faraci & Heistad, 1990) suggest that large intracranial arteries associated with the circle of Willis are key contributors to total cerebral vascular resistance. Also, evidence using high‐field magnetic resonance imaging (MRI) indicate that these vessels constrict with hypocapnia and dilate with hypercapnia (Coverdale, Lalande, Perrotta, & Shoemaker, 2015; Kellawan et al., 2016; Verbree et al., 2014). Therefore, large vessels contribute to CBF control. Of particular interest are the middle cerebral arteries (MCAs), which are the largest of the cranial vessels and distribute 80% of the blood flow to the brain (Nagata et al., 2016). Therefore, these vessels are the most studied with respect to cerebrovascular reactivity.

In young and healthy states, hypercapnia elicits an increase in CBF through complex mechanisms that include endothelial (Faraci & Heistad, 1998; Furchgott & Vanhoutte, 1989) and extravascular changes in pH (Kontos, Raper, & Patterson, 1977) and neuronal contributions to the vascular contractile state (Ainslie & Duffin, 2009; Toda, 1983). Of these mechanisms, nitric oxide (NO) represents a key endothelial factor stimulated by carbon dioxide (Hamel, 2004; Lavi, Egbarya, Lavi, & Jacob, 2003) affecting vascular tone. Although these effects are known for parenchymal vascular beds, the direct effect of NO on the basal cerebral arteries, and their upstream subcranial arteries (i.e. vertebral arteries and internal carotid arteries), remains unknown, particularly in humans.

Drugs such as sodium nitroglycerin (NTG) deliver an exogenous source of NO to large coronary and peripheral conduit vessels, where they cause dilatation (Arnold, Mittal, Katsuki, & Murad, 1977; Ignarro, Ross, & Tillisch, 1991; Parratt, 1979). When administered sublingually, this lipid‐soluble drug is rapidly absorbed over the mucosal membranes in the mouth (Zhang, Zhang, & Streisand, 2002). Sodium nitroglycerin has a half‐life of ∼2.5 min (Kirsten, Nelson, Kirsten, & Heintz, 1998b) and acts as a metabolism‐dependent NO donor, amplifying NO production and eliciting subsequent vasodilatory effect(s) (Kirsten et al., 1998b; Kirsten, Nelson, Kirsten, & Heintz, 1998a). Therefore, NTG is viewed as an ideal drug both for clinical use and for studying non‐endothelial vasomotor function (Mao et al., 2012). In addition, NTG can cross the blood–brain barrier and act directly on the vascular smooth muscle cells (independent of the endothelium), where it is assumed to biotransform to NO (Chen, Zhang, & Stamler, 2002). Based on observations in the brachial artery vascular bed, exogenous NO dilates conduit arteries some distance from the heart, but with little change in total blood flow and systemic blood pressure, owing to concurrent vasoconstriction (preventing dilatation) in the downstream vascular bed (Anderson, Meredith, Ganz, Selwyn, & Yeung, 1994; Ignarro et al., 1991). Consequently, NTG may provide a useful tool to study NO‐mediated control of large cerebral arteries. Despite its popular use in patients with cardiovascular disease, the role of NTG (and, therefore, NO) in the cerebrovasculature remains unstudied.

Therefore, this study tested the hypothesis that NO causes vasodilatation in the MCA of the circle of Willis. Additional focus on its blood flow provided insight into both the conduit vessel and the downstream vascular bed in response to exogenous NO.

## 2 METHODS

### 2.1 Ethical approval

Each participant was required to provide informed written consent to the protocols, which were approved by the Western University Health Sciences Research Ethics Board (HSREB: 107620). This study conformed to the standards set by the *Declaration of Helsinki* except for registration in a database.

### 2.2 Participants

Ten young, healthy individuals participated in this study (eight females, unknown menstrual phase; Table 1). Participants expressed no history of medication, cardiovascular disease or neurological disorder. Participants refrained from alcohol, caffeine and exercise for 12 h before testing.

**Table 1:** Participant characteristics

Males/females (*n*)	10, 2/8
Age (years)	22 ± 1
Height (cm)	171 ± 7
Body mass index (kg m^−2^)	21 ± 2
Systolic blood pressure (mmHg)	117 ± 13
Diastolic blood pressure (mmHg)	66 ± 7

Values are means ± SD.

### 2.3 Experimental protocol

Data were collected during two testing sessions that were performed on separate days: (i) a laboratory session; and (ii) a MRI session. In each session, measurements were made before and after NTG treatment (0.4 mg, Nitrolingual Pumpspray; Sanofi, Laval, QC, Canada) and separately during a placebo (PLO), composed of water and peppermint oil to match the flavour of NTG. Each treatment was provided via sublingual spray. All participants were blinded to treatment, and treatments were administered in a random order in the laboratory session that was then repeated in the MRI session.

#### 2.3.1 Laboratory session measurements

Height, weight and age were acquired from each participant (Table 1). Supine measurements included brachial artery diameter and blood velocity [duplex ultrasound machine with a 10 MHz probe (Vivid 7; GE Healthcare, Chicago, IL, USA)] and right MCA (rMCA) blood velocity [2 MHz transcranial Doppler (TCD) transducer; Neurovision, Multigon Industries, Elmsford, NY, USA]. The brachial artery data provided information about the systemic effects of NTG and PLO. Additional measurements included finger arterial blood pressure (Finometer; Finapres Medical Systems, Enschede, The Netherlands) corrected to brachial sphygmomanometric values, Finometer‐derived measures of stroke volume (SV) and cardiac output (CO), based on the Modelflow algorithm, heart rate (HR; three‐lead ECG) and partial pressure of end‐tidal carbon dioxide (P ET ,CO2; AD Instruments; Colorado Springs, CO, USA). All values were recorded at 1000 Hz using LabChart software (AD Instruments, Colorado Springs, CO, USA).

Following establishment of stable variables, 5 min of supine rest allowed for baseline (BL) measurements. During this time, two brachial artery images were captured at the beginning and end of the 5 min period. During NTG or PLO administration, collection of measurements occurred for 20 min each, and brachial artery images were captured every minute for the first 8 min to capture the peak effect of the drug, then at the 10th, 15th and 20th minute post‐treatment to follow recovery.

#### 2.3.2 MRI session measurements

All cerebrovascular anatomy measurements were performed on a 7 T neuro‐optimized MRI scanner (Siemens MAGNETOM; Erlangen, Germany) using an in‐house‐built eight‐channel transmit, 32‐channel head coil. Cross‐sectional area (CSA) measurements were made of the rMCA using a T1‐weighted 3D SPACE pulse sequence (Park, Mugler, Horger, & Kiefer, 2007) using a sagittal orientation, at high resolution (0.5 mm isotropic, whole brain), with the following imaging parameters: TE = 8.2 ms, TR = 700 ms, FOV = 224 mm, matrix = 448 × 448 × 256, turbo factor = 96, BW = 587 Hz/px, iPat = 2 and TA = 5:38 min. A radial trajectory minimized the echo time, as it is robust to motion and the distribution of artefacts (Glover & Pauly, 1992). First, a baseline T1 image (requiring 5–6 min to complete) was captured. Thereafter, a second and third T1 image were captured, commencing at 2 min after NTG or PLO administration to capture the period of peak dilatation. Of note, our laboratory demonstrated that MCA dilatation in response to hypercapnia develops progressively, with the peak response occurring at 3–4 min (Coverdale et al., 2015). Additionally, peak brachial dilatation in response to NTG occurs ∼3 min post‐spray (Ducharme, Dupuis, McNicoll, Harel, & Tardif, 1999). Therefore, we anticipate that our imaging parameters capture a time period of maximal NTG effect.

**Figure 1:**
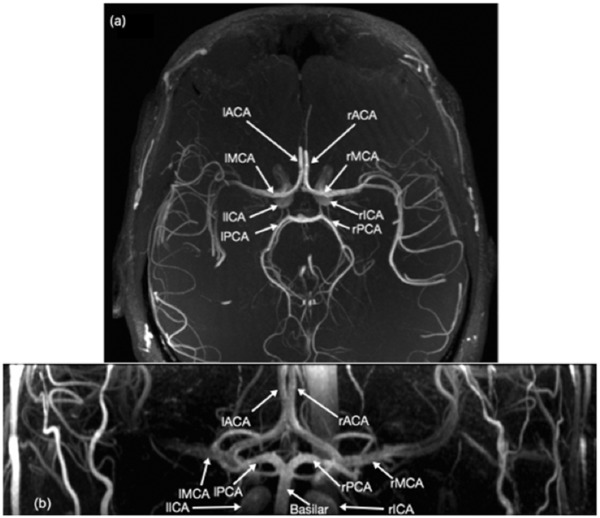
Time‐of‐flight magnetic resonance images of the circle of Willis at 7 T from a cranial (a) and a dorsal (b) view. Abbreviations: l/rACA, left/right anterior cerebral artery; l/rICA, left/right internal carotid artery; l/rMCA, left/right middle cerebral artery; and l/rPCA, left/right posterior cerebral artery

Thereafter, a time‐of‐flight (TOF) image was captured for structural/topological layout of the brain's conduit artery angiogram (Figure 1). As in the laboratory session, 20 min of recovery provided after each treatment allowed values to return to baseline. The P ET ,CO2 levels were monitored continuously via a modified oxygen mask (Trudell Medical Marketing Ltd, London, ON, Canada) with a built‐in sampling port, and a sampling line connected to a gas analyser (Biopac MP150; Biopac Systems Inc., Montreal, QC, Canada). All MRI images were saved as DICOM files for offline analysis.

### 2.4 Data analyses

**Figure 2:**
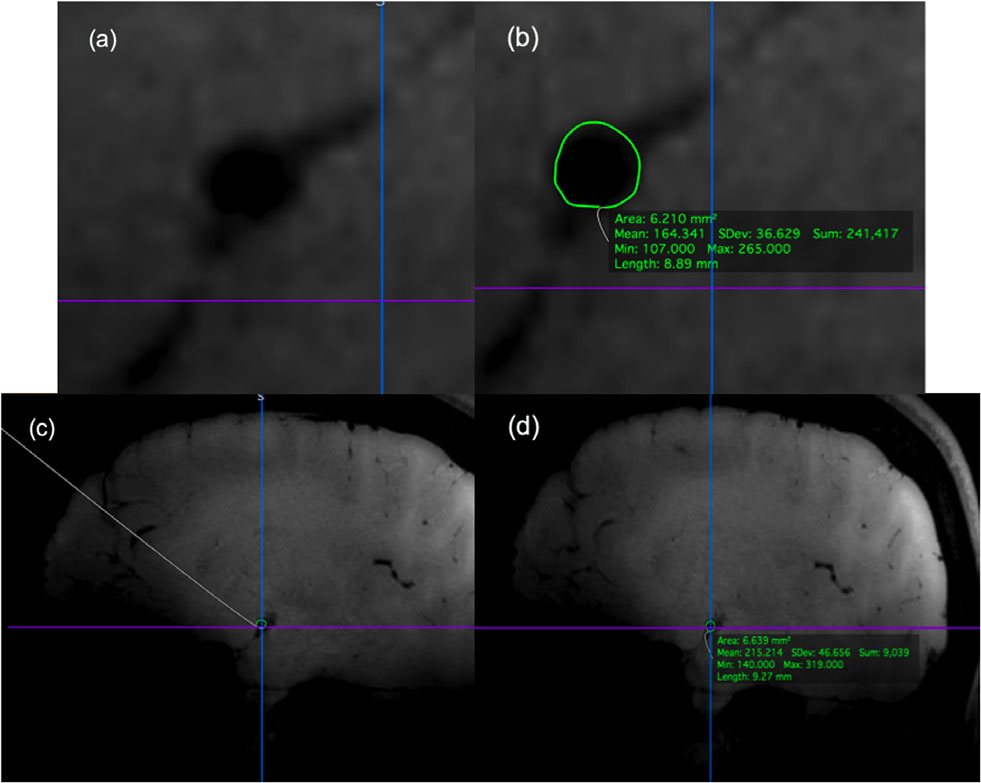
Right middle cerebral artery cross‐sectional area before (a) and after (b) measurement and comparison of a blinded baseline (c) and unknown treatment (d) of T1 images in Osirix

Laboratory measurements of CO, SV, HR, mean arterial pressure (MAP), P ET ,CO2, MCA velocity and brachial artery velocity, averaged over 30 s periods at every 1 min interval between 2 and 7 min post‐treatment, aligned with the T1 image acquisitions. Brachial artery CSA calculated from brachial artery diameter measurements (EchoPAC software; GE 7 Vingmed Ultrasound, Chicago, IL, USA) were taken every minute between 2 and 7 min post‐treatment. OsiriX software (Pixmeo SARL, Bernex, Switzerland) enabled measurements of the rMCA CSA in all three conditions, using previously described methods (Al‐Khazraji, Shoemaker, Gati, Szekeres, & Shoemaker, 2018). Anatomical location fiduciaries in the 3D images were obtained from the baseline images for each participant and condition. These anatomical locators, depth and contrast values enabled the location and measurement of vessel CSA at the same vascular site during the PLO and NTG treatments (Figure 2). In addition, two trained observers (J.M.S. and B.K.A.), blinded to treatment and participant information, measured CSA independently.

During the MRI, P ET ,CO2 was monitored to ensure stability, and averaged over 30 s obtained 2.5 min into the T1 scan (Table 2). The product of blood velocity (*V*) and CSA quantified blood flow in the MCA and brachial artery (flow = *V* × CSA). Conductance (*C*) for both the brachial artery and the MCA was also calculated as the quotient of flow and MAP (*C* = flow/MAP).

### 2.5 Statistical analysis

The effect of treatment on the rMCA CSA and haemodynamic outcomes was assessed using a one‐way repeated‐measures ANOVA, with a Bonferroni correction for multiple comparisons. The effects of treatment and time (each minute value) on MCA and brachial artery blood velocity data, normalized to baseline values, were assessed using a two‐way repeated‐measures ANOVA with a Bonferroni correction. A Bland–Altman test compared the blinded analyses of rMCA CSA from the two observers. The probability level for statistical significance was 0.05. Statistical analyses were performed in SPSS (SPSS 23; IBM Corporation, Armonk, NY, USA), and all data are presented as the mean ± SD.

## 3 RESULTS

### 3.1 Cross‐sectional area

**Figure 3:**
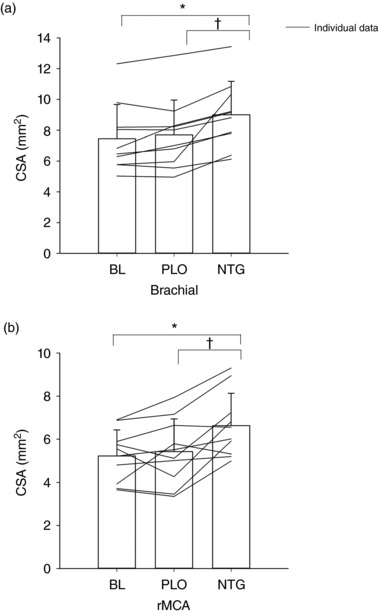
Changes in cross‐sectional area (CSA) in the brachial artery (a) and right middle cerebral artery (rMCA; b) during baseline (BL), placebo (PLO) and sodium nitroglycerin (NTG) treatments. ^*^NTG different from BL (*P *< 0.05). ^†^NTG different from PLO (*P *< 0.05)

All data passed a Sharipo–Wilk test of normality. Brachial artery CSA increased [*F*(2,18) = 13.92, *P *< 0.001] with NTG compared with both BL (9.0 ± 2.2 *versus* 7.5 ± 2.2 mm^2^, respectively, *P *< 0.01) and PLO (7.7 ± 2.3 mm^2^, *P *< 0.05), whereas measurements for BL and PLO treatments were similar (Figure 3a). The rMCA behaved in a similar manner [*F*(2,18) = 14.13, *P *< 0.001], in that CSA increased during NTG administration (6.6 ± 1.5 mm^2^) compared with both BL (5.2 ± 1.2 mm^2^, *P *< 0.001) and PLO (5.4 ± 1.5 mm^2^, *P *< 0.05); however, BL and PLO values were similar (Figure 3b). The Bland–Altman analysis of rMCA CSA between observers showed an inter‐rater bias of 0.6604 ± 1.075 mm^2^ (95% confidence interval 1.45–2.77) but no proportional bias. *Post hoc* power analysis (G*Power; Heinrich‐Heine‐Universität, Düsseldorf, Germany) for rMCA measurements was 0.99.

### 3.2 Blood velocity

**Figure 4:**
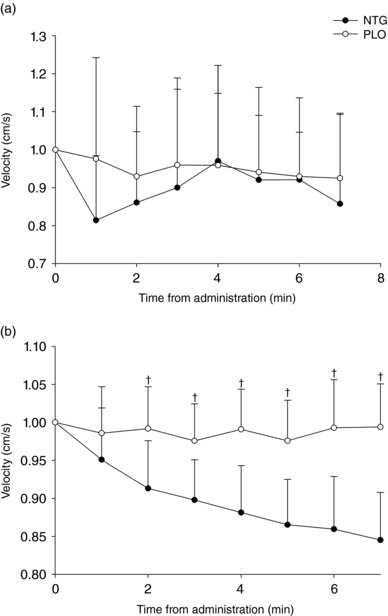
Velocity changes over 7 min of NTG and PLO treatments, normalized to baseline values for brachial artery (a) and right middle cerebral artery (rMCA; b). ^†^NTG different from PLO (*P *< 0.05)

Brachial artery velocity values did not change over time in either the NTG or PLO conditions (Figure 4a). However, a significant treatment × time interaction [*F*(2,18) = 87.21, *P *< 0.0001) indicated that MCA blood velocity during NTG was lower than during PLO at each time point after the first minute (Figure 4b; *P *< 0.05).

### 3.3 Other haemodynamic measurements

No effect of treatment was observed for MAP or P ET ,CO2 (Table 2). Heart rate increased [*F*(2,18) = 5.89, *P *< 0.05] during NTG compared with BL (66 ± 10 *versus* 62 ± 10 beats min^−1^, respectively, *P *< 0.05). Calculated total flow and vascular conductance in both brachial and rMCA vessels were similar to BL during NTG and PLO administration (Table 2).

**Table 2:** Haemodynamic parameters for baseline (BL), placebo (PLO) and nitroglycerin (NTG) conditions

Parameter	BL	PLO	NTG
Cardiac output (l min^−1^)	4.9 ± 1.9	4.8 ± 2.0	5.2 ± 1.7
Stroke volume (ml)	79 ± 32	76 ± 32	79 ± 28
Laboratory P ET ,CO2 (mmHg)	42 ± 4	42 ± 4	41 ± 5
MRI P ET ,CO2 (mmHg)	45 ± 6	45 ± 7	45 ± 7
Heart rate (beats min^−1^)	62 ± 10	64 ± 11	66 ± 10*
MAP (mmHg)	83 ± 12	86 ± 14	86 ± 13
rMCA *V* (cm s^−1^)	67 ± 10	62 ± 10‡	59 ± 9.3*
rMCA CSA (mm^2^)	5.2 ± 1.2	5.4 ± 1.5	6.6 ± 1.5*†
rMCA *F* (ml min^−1^)	221 ± 37.4	218 ± 35.0	213 ± 46.4
rMCA *C* (ml min mmHg^−1^)	2.7 ± 0.5	2.6 ± 0.8	2.6 ± 0.6
Brachial *V* (cm s^−1^)	3.0 ± 1.6	2.6 ± 1.9	2.8 ± 2.2
Brachial CSA (mm^2^)	7.5 ± 2.2	7.8 ± 2.3	9.0 ± 2.2*†
Brachial *F* (ml min^−1^)	14 ± 11	13 ± 12	16 ± 16
Brachial *C* (ml min mmHg^−1^)	0.2 ± 0.1	0.2 ± 0.1	0.2 ± 0.2

Values are means ± SD. Abbreviations: *C*, conductance; CSA, cross‐sectional area; *F*, flow; MAP, mean arterial pressure; P ET ,CO2, partial pressure of end‐tidal carbon dioxide; rMCA, right middle cerebral artery; and *V*, velocity. ^*^NTG different from BL (*P *< 0.05). ^†^NTG different from PLO (*P *< 0.05). ^‡^PLO different from BL (*P *< 0.05).

## 4 DISCUSSION

The major findings of this study are as follows: (i) the rMCA dilated in response to NTG administration; and (ii) despite an increase in CSA, there were no changes in overall flow, in both the MCA and brachial artery vascular beds. Therefore, exogenous NO dilates the large conduit basal cerebral arteries, but not the downstream vascular beds, in both brachial and rMCA vascular beds.

Sodium nitroglycerin is a common drug used to treat angina pectoris through its effect on dilatation of both large arteries and veins (Parratt, 1979). This, in turn, reduces both cardiac afterload and preload, respectively (Ludbrook, Byrne, Kurnik, & McKnight, 1977), with little change in blood pressure (Gori, Floras, & Parker, 2002; Nyberg & Westling, 1981). These changes appear to be mediated by concurrent increases in HR, and a blunting of baroreflex sensitivity facilitates increasing sympathetic contributions to cardiovascular control (Gisolf et al., 2004; Gori et al., 2002).

In this study, the expected brachial artery vasodilatation occurred during NTG (Anderson et al., 1994; Ignarro et al., 1991). A similar dilatory effect of ∼24% was observed in the rMCA perfusing the brain. Unexpectedly, the PLO also caused a small dilatory response, accounting for ∼4% of the change of CSA. Possible explanations for the PLO‐induced dilatation include a genuine placebo effect, or random sampling error and/or variability owing to the small sample size. Another explanation might be that the peppermint oil that forms a component of the NTG treatment was added to water in this study to produce a similar taste between the PLO and NTG treatments. Sodium nitroglycerin includes 1% menthol because it potentiates dilatory effects (Busiashvili, 2003). Peppermint oil contains menthol, an agonist for transient receptor potential melastatin 8 (TRPM8; Johnson et al., 2009). This receptor elicits dilatation in large arteries, such as the aorta (Johnson et al., 2009). Menthol also attenuates vasoconstriction in the mesenteric arteries (Sun et al., 2014). We speculate that these actions might also occur in the cerebral vessels. Regardless, the small (∼4 ± 19%) dilatation with PLO does not account for the much larger (∼28 ± 15%) MCA dilatation after NTG, leaving ∼24% attributed to exogenous NO. Additionally, we subtracted the CSA of PLO from NTG, and added that value to the BL values to determine the effect of exogenous NO. Using Student's two‐tailed paired *t* test, *P *< 0.05, indicating that exogenous NO significantly increases CSA.

The observation that exogenous NO causes cerebral artery dilatation is novel in humans although these data are preceded by earlier studies in rodents, in which exogenous NTG caused vasodilatation in the large intracranial arteries (Kistler, Vielma, & Davis, 1982). Furthermore, single photon emission computed tomography measurements of CBF and TCD measurements of MCA blood velocity (to estimate changes in diameter) indicated an increase in calculated MCA diameter after NTG administration (Dahl, Russell, Nyberg‐Hansen, & Rootwelt, 1989). The present study confirms these earlier studies, with the first direct measurements of the MCA during NTG administration in healthy adults. The values of P ET ,CO2 before and during NTG were similar in both the MRI and the laboratory, indicating that the MCA response to NTG occurred independently of any confounding sources of dilatation elicited by hypercapnia. Of note, the changes in rMCA CSA observed in the present study (5.2–6.6 mm^2^) after NTG are similar to those provided earlier in response to hypercapnia in different individuals (5.6–6.5 mm^2^; Coverdale, Gati, Opalevych, Perrotta, & Shoemaker, 2014).

This study indicates an important role for exogenous delivery of NO in cerebral conduit vessel control. However, the role of endogenous NO in conduit vessel dilatation remains equivocal. In rats, pharmacological blockade of NO synthase (NOS) attenuated the increase in CBF during hypercapnia, demonstrating the role of NO in cerebrovascular dilatation (Wang, Paulson, & Lassen, 1992). In contrast, human studies of CBF control using NOS inhibition have produced conflicting results; some studies reported little effect of NOS on total CBF during reactive hypercapnic states (Ide, Worthley, Anderson, & Poulin, 2007), whereas others reported a blunted increase in MCA blood velocity with hypercapnia after NOS inhibition (Schmetterer et al., 1997). A major difference between these earlier studies in humans is that the former measured total flow, whereas the latter measured blood velocity, not accounting for conduit artery CSA. Previously, researchers in our laboratory (Coverdale et al., 2014) and others (Verbree et al., 2014) demonstrated with high‐field MRI that measurement of blood velocity underestimates the changes in CBF during hypercapnia and hypocapnia. Therefore, the discrepancy regarding the effect of NO on CBF control can be resolved with conduit artery CSA measurements. In this perspective, the combined data suggest that NO has a specific role in dilating basal cerebral and subcranial arteries.

Measurements of both MCA CSA and flow velocity provide insight into the effect of NTG on both the conduit artery and the downstream vascular bed. Thus, a second major outcome of this study was the observation that, despite the increase in CSA after NTG and stable MAP, blood velocity decreased in the MCA such that overall CBF remained unchanged. This observation supports previous findings of reductions in MCA blood velocity after sublingual NTG administration (Dahl et al., 1989; Zuj et al., 2012). Likewise, others observed diminished MCA blood velocity but little change in regional CBF after intravenous infusions of sodium nitroglycerin (Iversen, Holm, Friberg, & Tfelt‐Hansen, 2008; White et al., 2000). The authors of these previous studies hypothesized that MCA dilatation might occur, providing a possible explanation for the apparently disparate observations. The present study provides the data needed to resolve this uncertainty. By quantifying dilatation in this large conduit vessel, as well as a reduction in blood velocity after sublingual NTG, we conclude that exogenous NO affects dilatation in the rMCA but not in the downstream vascular beds.

The mechanism(s) mediating the lack of downstream dilatation with NTG remains speculative. The lack of a change in cerebral and brachial blood flow despite conduit artery dilatation and similar values of MAP suggests no change in downstream vascular resistance. Despite known sensitivity of parenchymal vessels to NO (Anderson et al., 1994; Ignarro et al., 1991; Lavi et al., 2003), the observation of similar vasomotor responses in the vascular beds of the MCA and brachial arteries suggests a similar underlying physiology. One possible mechanism could be a sympathetic nervous system response that has proportionately greater effects in the downstream *versus* conduit vessels, thereby competing with the dilatory effect of the delivered NO. Previously, it has been reported that NTG leads to a reflexive increase in plasma noradrenaline to counter the drug‐induced hypotension (Tassorelli, Blandini, Costa, Preza, & Nappi, 2002). Other *in vivo* studies in the rat and guinea‐pig determined that intravenous NTG administration induced increases in noradrenaline, mimicking biological responses associated with sympathetic neuronal activity in perfused atria and cerebrospinal fluid (Ma & Long, 1991a, b, c; Ma, Schmid, & Long, 1994; Tassorelli et al., 2002). In humans, chronic NTG administration decreased baroreflex sensitivity and increased sympathetic neural modulation of heart rate (Gori et al., 2002). Furthermore, acute reflexive sympathetic vasoconstriction in the cerebral circulation has been difficult to observe in the human brain in conditions of stable blood pressure (Sándor, 1999), although small effects have been observed using a lower‐body negative pressure suction model (Wilson, Serrador, & Shoemaker, 2003). Thus, although the literature remains uncertain of the magnitude of sympathetic activation in the control of CBF (Brassard, Tymko, & Ainslie, 2016), neurogenic vasoconstriction might be one potential compensatory mechanism to counter the drug‐induced dilatation.

Limitations of this study include the collection of MRI and laboratory measurements on different days owing to MRI availability. However, existing data suggest that this effect might be negligible. For example, TCD estimates of flow velocity are reproducible for up to 1 week (Demolis, Chalon, & Giudicelli, 1993), and phase‐contrast MRI analyses for estimating CBF are reproducible over 72 days (Spilt et al., 2002). Furthermore, a single‐subject study demonstrated that over 20 consecutive days, measurement of velocity in the MCA using TCD is reliable (Vingerhoets & Stroobant, 2002). Additionally, previous studies in our laboratory demonstrated reproducibility of CBF velocity measurements on different days (TCD and phase‐contrast MRI), reporting an Intraclass correlation (ICC) of 0.83 (*P* < 0.001; Coverdale et al., 2015). Therefore, the data suggest that CBF measurements are similar on different days. To address this issue further, a multiple regression of the present results demonstrated that changes in MCA flow velocity occurred independently of changes in MAP, CO and P ET ,CO2 [*F*(3,6) = 0.382, *P *= 0.77, *R* ^2 ^= 0.4].

A second limitation of the present study is that an increase in baseline P ET ,CO2 in the MRI *versus* the laboratory might have induced different baseline MCA CSA and therefore, responses to NTG. Additionally, Battisti‐Charbonney, Fisher, & Duffin (2011) demonstrated a sigmoidal relationship of increasing CO_2_ values and MCA velocity below a discernable threshold (where above 50 mmHg, the relationship changes to linear changes in velocity). Between 40 and 50 mmHg, the increase in velocity remains relatively linear. We do not yet know the CSA–P ET ,CO2 dose–response relationship, but the values we obtained occurred on the linear part of the velocity–P ET ,CO2 curve; therefore, we can infer that the difference between the two testing sessions may be small. To address this possibility, we evaluated changes in CSA, although this may not directly address the problem of baseline CSA affecting the responsiveness of the MCA to exogenous NO.

A third possible limitation is the time between treatment administrations and the lack of a second baseline measurement before the second treatment. As mentioned, the half‐life of NTG in plasma is ∼2.5 min (Kirsten et al., 1998b). Seven half‐lives were allowed after the NTG treatment to optimize the loss of drug effects. Furthermore, haemodynamics, velocity levels in the MCA and brachial artery, and the vessel diameters indicated reinstitution of baseline conditions between PLO and NTG conditions. Also, the order of treatments was randomized to ameliorate the potential problem of treatment carryover effects. Maximal vasodilatation in the brachial artery is observed 2 min post‐NTG administration (Ducharme et al., 1999), becoming barely detectable after 20 min (Armstrong, Armstrong, & Marks, 1979). Therefore, physiological measures suggested that a return to baseline conditions had occurred after 20 min, although some residual but undetectable NTG effects might have persisted in some individuals in whom NTG was given before PLO.

### 4.1 Conclusion

The rMCA dilated in response to sublingual 0.4 mg NTG, suggesting an NO‐mediated mechanism. This dilatation did not change total flow because of concurrent reductions in blood velocity, suggesting differential drug effects on conduit arteries *versus* microvascular territories.

## COMPETING INTERESTS

None declared.

## AUTHOR CONTRIBUTIONS

J.M.S. and B.K.A. collected and analysed the data. All authors took part in the conception and design and in drafting and revising the manuscript. All authors approved the final version of the manuscript and agree to be accountable for all aspects of the work in ensuring that questions related to the accuracy or integrity of any part of the work are appropriately investigated and resolved. All persons designated as authors qualify for authorship, and all those who qualify for authorship are listed.
